# Interactive Effects of Light Intensity and Nitrogen Supply on Shoot Emergence and Associated Photosynthetic Traits in *Dendrocalamus latiflorus*

**DOI:** 10.3390/biology15010049

**Published:** 2025-12-27

**Authors:** Jundong Rong, Jiaying Liu, Heng Lei, Yuchen Lin, Jiawei Wang, Qiulan Guo, Azra Seerat, Tianyou He, Liguang Chen, Yushan Zheng, Lili Fan

**Affiliations:** 1 College of Forestry, Fujian Agriculture and Forestry University, Fuzhou 350002, China; 2 College of Landscape Architecture and Art, Fujian Agriculture and Forestry University, Fuzhou 350002, China; 3 Research Institute of Subtropical Forestry, Chinese Academy of Forestry, Hangzhou 311400, China

**Keywords:** *Dendrocalamus latiflorus*, optical light gradient, bamboo shoot germination, coupling effects, shooting stage regulation

## Abstract

Investigating light–nitrogen interactions during bamboo shoot bud germination of *Dendrocalamus latiflorus* provides a theoretical basis for regulating shoot emergence through optimized light intensity and nitrogen supply. This study further elucidates how such coupling effects can guide stand density adjustments in bamboo plantations. Additionally, it provides actionable insights for improving nutrient management strategies in practical bamboo cultivation systems. This study evaluated the effects of different nitrogen levels (1.5, 4.5, and 7.5 g·clump^−1^) combined with varying light intensities (10%, 40%, and 100%) on shoot germination and physiological characteristics of bamboo. Two-way ANOVA revealed a significant interaction between light intensity and nitrogen concentration on bamboo shoot bud germination (*p* < 0.05). Under low nitrogen conditions, bamboo shoot bud germination was highest (3 buds·clump^−1^) at 10% light intensity. However, under high nitrogen conditions, the highest germination (4.75 buds·clump^−1^) occurred at 40% light intensity. Moreover, leaf carbon, nitrogen, and starch contents were positively correlated with shoot bud germination. Pearson correlation and path analyses indicated that total chlorophyll, chlorophyll a/b, photochemical quenching coefficient, sucrose, and leaf nitrate were the main photosynthetic traits associated with bamboo shoot bud germination. Among these, the photochemical quenching coefficient was the most critical factor. Based on this, the nitrogen management, nitrogen utilization, and low-light adaptability of *D. latiflorus* could be optimized.

## Simple Summary

Fertilization and light are the key factors affecting the development of plant lateral buds. Therefore, maintaining the optimal balance of light and nitrogen is essential to promote plant growth. A key objective of research in this field is to provide practical guidance for nutrient management in bamboo cultivation systems. Our study found that chlorophyll, chlorophyll a/b, photochemical quenching, sucrose, and leaf nitrate were the main photosynthetic characteristic factors, and photochemical quenching was the most important internal regulatory factor. From the perspective of the development stage, nitrogen fertilizer plays a dominant role in promoting bud germination at the early stage of germination; in the middle stage of seedling emergence, light intensity and nitrogen significantly affected the development of new shoots. In the later period, the light intensity was the dominant factor. Thus, balanced regulation of light and nitrogen supply in the understory during the shooting period can effectively enhance shoot germination and growth in *Dendrocalamus latiflorus*.

## 1. Introduction

*Dendrocalamus latiflorus* is one of the key bamboo species cultivated in southern China due to its significant economic, ecological, and social value. However, commercial bamboo forests face challenges such as intensive management practices and low productivity, which hinder the optimal production of bamboo shoots. Addressing these issues is crucial for improving the overall yield and quality of bamboo shoots. Research has shown that shading can enhance the germination of *D. latiflorus* shoots. Specifically, seedlings exhibit greater light-use efficiency (LUE) under 20% (100 μmol·m^−2^·s^−1^) to 30% (150 μmol·m^−2^·s^−1^) light conditions, which promotes higher photosynthetic rates [1]. Additionally, moderate nitrogen fertilization has been found to benefit the growth of *D. latiflorus* seedlings. For instance, Lin et al. [2] observed that a nitrogen application rate of 22.5 g·clump^−1^ optimally balanced carbon and nitrogen metabolism, enhancing the photosynthetic capacity of the plant. Despite these findings, most research has focused on either light or nitrogen in isolation. There is a clear need for more comprehensive studies investigating how the interaction between light intensity and nitrogen supply regulates bamboo shoot growth and adaptation. Understanding these mechanisms will provide valuable insights for optimizing fertilization and shading strategies, ultimately improving the productivity and sustainability of *D. latiflorus* cultivation.

Fertilization is a key factor affecting the development of lateral buds, with nitrogen playing a crucial role in promoting lateral growth [3]. Studies have shown that varying nitrogen concentrations influence the growth of axillary buds, which impacts tillering and branch development [4,5]. Previous studies have shown that light intensity significantly affects the accumulation and distribution of photosynthetic products in various crops, thereby regulating tillering and yield formation [6,7,8]. Although these studies have primarily focused on crops, their conclusions provide a foundation for exploring the role of light regulation in the development of lateral buds in bamboo. Nitrogen and light are interrelated factors in plant growth. Research shows that with increasing light intensity up to a certain threshold, the photosynthetic rate increases, leading to a higher nitrogen demand [9]. However, when soil nitrogen is limited, plants must balance their physiological responses to the available light and nitrogen. For instance, increasing nitrogen supply under low light conditions can improve photosynthesis in *Tilia amurensis* and *Phoebe zhennan*, while excessive nitrogen can inhibit this process [10,11]. Maintaining an optimal balance between light and nitrogen is essential for promoting plant growth. Although previous studies have explored the individual effects of these factors, few have focused on their combined impact on lateral bud development. Investigating this interaction is crucial for optimizing fertilization strategies, improving field production efficiency, and mitigating low-light stress on lateral bud growth through nitrogen application.

The lateral bud differentiation and shoot development of bamboo plants are key to bamboo forest regeneration and artificial breeding. The germination rate of bamboo shoots is influenced not only by factors such as provenance, age, and physiological state, but also by external environmental conditions [12]. Studies have shown that the germination process of shoot buds mainly includes three stages: the activation of dormant axillary buds, the release of apical dominance, and the elongation of new buds [13]. Environmental factors such as light, temperature, water, and nutrient supply can significantly regulate the physiological activities of shoot buds; especially light intensity and nitrogen level play a key role in regulating carbon and nitrogen metabolism, hormone balance, and energy distribution of buds [12]. At present, it has been reported that moderate light intensity can promote the rooting and germination of *Phyllostachys edulis* shoots and improve the survival rate of seedlings [14]. In addition, exogenous nitrogen can promote the accumulation of soluble protein and chlorophyll in shoot buds, enhance photosynthetic capacity, and thus increase germination rate [15]. However, most of the existing research objects are single varieties such as *P. edulis*. The research on the synergistic regulation of light and nitrogen during the germination of *D. latiflorus* shoots is still insufficient, which limits the optimal application of light intensity management and fertilization ratio in actual cultivation. Therefore, it is of great theoretical and practical significance to systematically explore the interaction between light intensity and nitrogen supply and its effect on shoot germination and early growth of *D. latiflorus*.

In this study, we investigated the germination and physiological characteristics of *D. latiflorus* shoots under three nitrogen application levels (1.5, 4.5, and 7.5 g·clump^−1^) and varying light intensities (10% (50 μmol·m^−2^·s^−1^), 40% (200 μmol·m^−2^·s^−1^), and 100% (500 μmol·m^−2^·s^−1^)). The study hypothesized that there was a significant interaction between different light intensities and nitrogen levels on seedling germination and nitrogen absorption and utilization. We also analyzed how nitrogen absorption and utilization were influenced by these light and nitrogen conditions. By examining the combined effects of light intensity and nitrogen levels, this research provides empirical data to guide optimal shoot germination. The findings offer practical insights for regulating light and nitrogen in bamboo cultivation, contributing to improved nutrient management and adjustments in stand density for more efficient *D. latiflorus* shoot production.

## 2. Materials and Methods

### 2.1. Study Site

The experiments were conducted in the greenhouse at the College of Forestry, Fujian Agriculture and Forestry University, Fuzhou, China (119°13′51.18″ E, 26°05′4.35″ N). The greenhouse was well-ventilated and equipped with spray cooling systems and shading nets. During the experiment, temperatures ranged from 14 °C to 30 °C, with an average of 25 °C. The relative humidity was above 78%, the natural photoperiod was approximately 12 h, from 6:00 to 18:00.

### 2.2. Study Design

**Figure 1:**
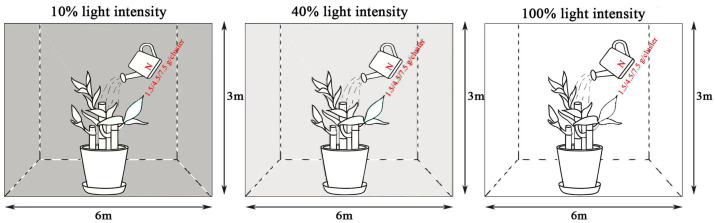
A schematic diagram of different treatments. (This Figure is a schematic diagram of the experimental layout and is not drawn to scale).

This study utilized a completely randomized design with factorial arrangements of light intensity and nitrogen levels. A gradient of light intensity was achieved by installing sunshades with varying transmittances (100%, 40%, and 10%). Light intensities for each treatment were measured at noon on a sunny day using a photon sensor (LI-190R, LI-COR, Lincoln, NE, USA) and were approximately 500, 200, and 50 μmol·m^−2^·s^−1^, respectively (Figure 1). Light conditions remained largely stable throughout the experiment, and the shading effectively controlled light variations between treatments, meeting the experimental requirements for light gradients. Pots were spaced to avoid mutual shading among plants. Although previous studies have reported relatively high light use efficiency at intermediate light levels (e.g., 20–30% light intensity), the light treatments in this study were designed to represent contrasting and management-relevant light environments rather than to identify a narrow photosynthetic optimum. Accordingly, severe shading (10%), moderate shading (40%), and full light (100%) were selected to capture light-limited, typical understory, and open-canopy conditions in bamboo cultivation systems.

Three nitrogen concentration gradients were applied: 1.5 g·clump^−1^ (N1, low nitrogen), 4.5 g·clump^−1^ (N2, medium nitrogen), and 7.5 g·clump^−1^ (N3, high nitrogen). In this study, nitrogen application rates were expressed on a per-clump basis. One bamboo clump refers to a group of shoots sharing the same rhizome system and planted in a single pot, which served as an independent experimental unit. Each bamboo clump was grown in an individual pot. All pots used in the experiment were identical in size and material and were equipped with drainage holes at the bottom. Pot characteristics were kept constant across all treatments and were not considered as experimental variables. A uniform growth substrate was used for all plants. The substrate was thoroughly mixed prior to planting to ensure homogeneous physical and chemical properties among treatments. Substrate conditions were consistent throughout the experiment and were not manipulated as part of the experimental design.

Nitrogen was applied to the soil through irrigation using urea (46% N), rather than by foliar spraying. The reported nitrogen rates represent the total amount of nitrogen applied per clump during the entire experimental period. Nitrogen application rates were determined based on conventional fertilization levels in different bamboo cultivation management systems. Nitrogen treatments were applied simultaneously under the three light intensities described above, with treatment durations consistent with the light experiment. Nine treatment combinations (three light levels × three nitrogen levels) were tested. The total nitrogen amount was divided into three applications at 30% (I), 40% (II), and 30% (III) (Table 1). Each treatment had eight replicates, with one clump of *D. latiflorus* planted in each pot.

Urea (46% nitrogen), calcium superphosphate (12% P_2_O_5_), and potassium chloride (60% KCl) were used as fertilizers. The nitrogen fertilizer dosage varied according to the experimental treatments, while phosphorus fertilizer was applied uniformly at 6 g·clump^−1^. Potassium chloride was applied as a base fertilizer at 1.5 g·clump^−1^ during the first fertilization. For the nitrogen application, urea was dissolved in water and applied through irrigation. Phosphorus and potassium fertilizers were applied manually by creating a ring-shaped trench 10 cm deep and 10 cm away from the bamboo stump. The shading experiment began on 10 April 2019. Fertilization was applied one month after the shading commenced, on three dates: 10 May, 10 July, and 31 August 2019. Bamboo shoot emergence in *D. latiflorus* followed three typical growth stages in Fujian Province: the early stage (early to mid-June), the peak stage (mid-June to mid-August), and the late stage (mid-August to early October).

Shoot emergence was recorded during each of these three periods. Additionally, photosynthetic pigments, gas exchange parameters, chlorophyll fluorescence, carbohydrate content in the leaves, as well as carbon and nitrogen levels in the leaves were measured at three time points: mid-June, mid-August, and early October. Throughout the experiment, the potted *D. latiflorus* was maintained by regular weeding, insecticide application, and the removal of excess shoots. The experiment was conducted under open or semi-open conditions, where plants were exposed to natural rainfall. No additional rain-shading measures were applied. To minimize potential interference from rainfall, all pots were managed uniformly, and drainage was ensured to prevent waterlogging. Water and nutrient management were kept consistent across treatments so that rainfall did not differentially affect the experimental treatments.

### 2.3. Investigation of the Number of Emerged Bamboo Shoot Buds

From the start of application of nitrogen treatment, the growth of shoot buds (defined as shoot tips less than 5 cm in length) was monitored. The number of shoot buds emerging during each defined shooting period was recorded. After each period of investigation, newly emerged shoots were cut off to prevent the development of new bamboo culms. Only the final group of shoots at the end of the shooting was retained. In this experiment, eight pots per treatment were used as samples rather than independent replications.

### 2.4. Determination of Photosynthetic Characteristics of Leaves

#### 2.4.1. Determination of Photosynthetic Pigments

Mature leaves were selected from the upper, middle, and lower parts of bamboo culms from each clump. After being washed with clean water and dried, the leaves had their midribs removed, and the leaves were cut and mixed. A 0.20 g sample of mixed leaves was weighed. Chlorophyll was measured following the direct extraction method [16]. The contents of chlorophyll a (Chl a), chlorophyll b (Chl b), total chlorophyll (Chls), and carotenoid (Car) were calculated using the formulas described by Lichtenthaler et al. [17]. Ratios of Chl a/b and Car/Chl ratios were also calculated. Each treatment had four replicates. The formula is as follows:

Chl a (mg·g^−1^) = (12.7D663 − 2.69D645) × V/(1000 × W) (1)

Chl b (mg·g^−1^) = (22.9D645 − 4.68D663) × V/(1000 × W) (2)

Chls (mg·g^−1^) = (20.2D645 + 8.02D663) × V/(1000 × W) (3)

Chl a/b (mg·g^−1^) = Chla/Chlb (4)

Car (mg·g^−1^) = (1000D470 − 3.27Chla − 104Chlb) × V/(1000 × W × 229) (5)

Car/Chls (mg·g^−1^) = Car/Chls (6)

#### 2.4.2. Determination of Gas Exchange Parameters

Mature functional leaves from the middle and upper parts of bamboo culms were selected from each clump for measurement. A leaf chamber (2 × 3 cm) with red and blue light sources was used, and the measurements were taken under a light saturation light intensity of 1600 μmol·m^−2^·s^−1^. Before measurement, the leaves were induced under the same light intensity for 20–30 min. Each treatment involved four replicates. The net photosynthetic rate (P_n_), intracellular CO_2_ concentration (C_i_), transpiration rate (T_r_), stomatal conductance (g_s_), and water use efficiency (WUE) were measured using a Li-6400 XT portable photosynthesis system (LI-COR, Lincoln, NE, USA). WUE was calculated as P_n_/T_r_ [18].

#### 2.4.3. Determination of Chlorophyll Fluorescence Characteristics

Chlorophyll fluorescence parameters, including actual photochemical efficiency (Φ_PSII_), apparent photosynthetic electron transport rate (ETR), photochemical quenching coefficient (qP), and non-photochemical quenching coefficient (NPQ), were measured using an OS5p portable pulsed modulated chlorophyll fluorescence analyzer (OPTI-sciences, Hudson, NH, USA). After 30 min of dark adaptation, the leaves were exposed to natural light for 5–7 min before determining PSII photochemistry (F_v_/F_m_) [19]. Each treatment involved four replicates.

### 2.5. Determination of Sucrose and Non-Structural Carbohydrates in Leaves

For each treatment, upper, middle, and lower mature leaves were collected from different bamboo culms, mixed, and washed with ultrapure water. After removing the midribs, the leaves were chopped and combined. A 0.10 g sample was used to determine the contents of sucrose, starch (ST), and soluble sugar (SS). These measurements were used to calculate the total non-structural carbohydrate (NSC = soluble sugar + starch) content and the SS/ST ratio [20]. All indicators were analyzed using physiological and biochemical kits (Suzhou Keming Biotechnology Co., Ltd., Suzhou, China). Four replicate pots were measured per treatment.

### 2.6. Determination of Leaf Carbon and Nitrogen Characteristics

Leaves: Upper, middle, and lower mature leaves were collected, washed with ultrapure water, cut, and divided into two parts for analysis: Fresh samples (0.10 g) were analyzed for nitrate nitrogen (LNO_3_^−^-N) and ammonium nitrogen (LNH_4_^+^-N) using a plant nitrogen kit. Remaining samples were dried at 105 °C for 15 min, then at 85 °C until constant weight. Ground and sieved samples (0.25 g) were analyzed for total nitrogen (LN) and total carbon (LC) using a carbon-nitrogen analyzer, and the LC/LN ratio was calculated [21].

### 2.7. Data Processing and Analysis

Data were analyzed using Excel 2016, SPSS 22.0, and R 4.4.3 software. Two-way ANOVA was performed to test the effects of light intensity and nitrogen levels on all measured indicators. One-way ANOVA was used to assess the significance of each index during different shooting periods (α = 0.05). Pearson correlation analysis was conducted to analyze the relationship between shoot bud germination and leaf physiological and biochemical indices under different light and nitrogen treatments. Path analysis was performed using the ‘lavaam’ and ‘semTools’ packages in R 4.4.3 software to explore the combined effects of leaf functional traits on shoot germination rate. Graphs and tables were generated using Excel 2016 and Origin 2024. In this factorial design, nitrogen (N) and light intensity (l) were treated as two fixed factors, and one-way ANOVA was applied for graphical comparisons of nitrogen treatments within the same light intensity (*p* < 0.05).

## 3. Results

### 3.1. Bud Germination Across Shooting Stages

**Figure 2:**
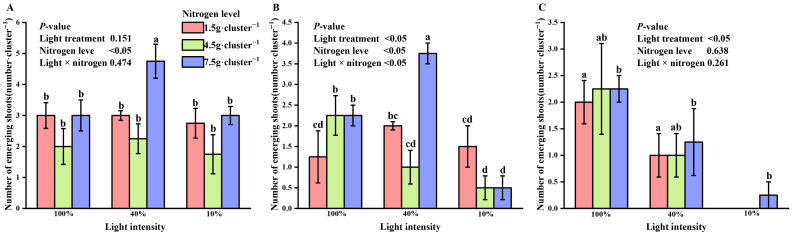
Interaction effects of light intensity and nitrogen concentration on shoot germination of *Dendrocalamus latiflorus* seedlings. (Values are presented as means ± standard deviation (SD). Different letters indicate significant differences among nitrogen treatments within the same light intensity (one-way ANOVA, *p* < 0.05). (**A**), initial shooting phase; (**B**), intermediate shooting phase; (**C**), late shooting phase; 1.5 g·clump^−1^, nitrogen level was 1.5 g·clump^−1^; 4.5 g·clump^−1^, nitrogen level was 4.5 g·clump^−1^; 7.5 g·clump^−1^, nitrogen level was 7.5 g·clump^−1^).

At the early stage of shooting, nitrogen treatment had a significant effect on the number of new shoots (*p* < 0.05). Under high nitrogen conditions, the number of new shoots was significantly greater at 40% light intensity than at 10% or 100% light intensity, as indicated by two-way ANOVA (Figure 2A).

In the middle stage of shoot emergence, both light and nitrogen treatments, as well as their interaction, significantly influenced the number of new shoots. At 40% light intensity, the number of new shoots was higher under low and high nitrogen treatments compared to 10% and 100% light intensities. Under low and medium nitrogen conditions, 10% light intensity led to more new shoots than 100% light, but this trend reversed as nitrogen levels increased (Figure 2B).

By the end of shoot emergence, two-way ANOVA showed that light intensity (l), nitrogen treatment (N), and their interaction (N × l) significantly affected the number of new shoots (*p* < 0.05). The highest number of new shoots was observed under 100% light intensity compared to 10% and 40% light. High nitrogen treatment continued to promote shoot formation, whereas low and medium nitrogen treatments had no significant impact (Figure 2C). High nitrogen treatment was associated with higher numbers of new shoots at the early and late stages of germination. The highest shoot numbers were observed at 40% light intensity in the early stage and at 100% light intensity in the late stage. A stage-dependent reversal in the interaction between light intensity and nitrogen was observed during the middle shooting stage.

### 3.2. Photosynthesis in Leaves During Shooting

#### 3.2.1. Photosynthetic Pigments in Leaves During Shooting

**Figure 3:**
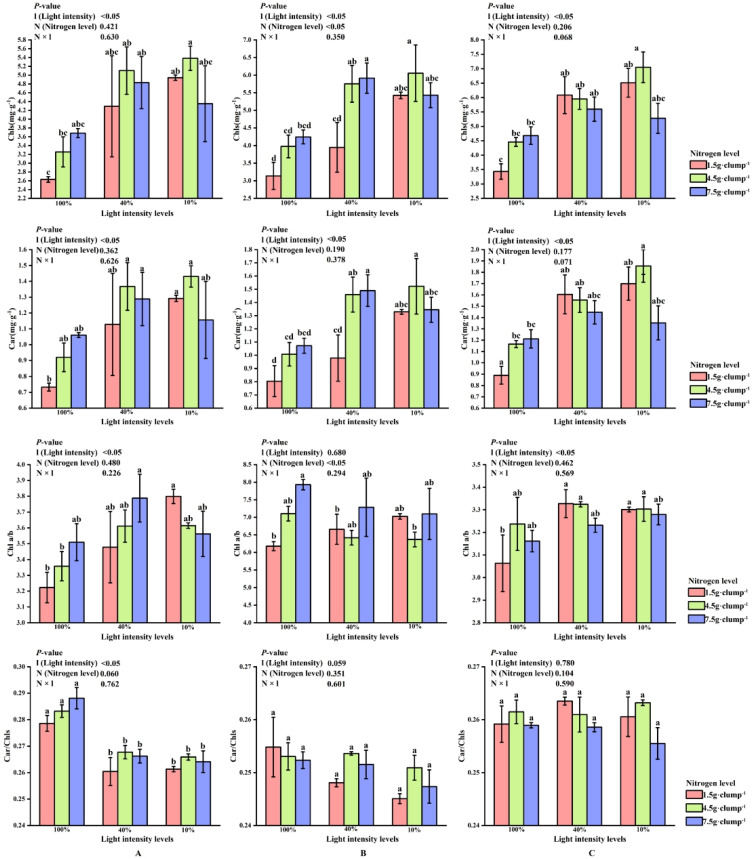
Interaction effects of light intensity and nitrogen concentration on photosynthetic pigments in *Dendrocalamus latiflorus* seedlings. (Values are presented as means ± standard deviation (SD). Different letters indicate significant differences among nitrogen treatments within the same light intensity (one-way ANOVA, *p* < 0.05). (**A**), initial shooting phase; (**B**), intermediate shooting phase; (**C**), late shooting phase; 1.5 g·clump^−1^, nitrogen level was 1.5 g·clump^−1^; 4.5 g·clump^−1^, nitrogen level was 4.5 g·clump^−1^; 7.5 g·clump^−1^, nitrogen level was 7.5 g·clump^−1^. Abbreviations: Car, carotenoids; Chls, total chlorophylls; Chl a/b, chlorophyll a/b; Car/Chls, carotenoids to total chlorophylls).

Two-way ANOVA showed that light intensity (l), nitrogen treatment (N), and their interaction (N × l) significantly affected leaf photosynthetic characteristics (*p* < 0.05). In the early shooting stage, light intensity significantly affected the contents of Chls, Car, and the ratios of Chl a/b and Car/Chls (*p* < 0.05). Under high nitrogen treatment, Chl and Car contents were highest at 40% light intensity, followed by 10% light intensity (Figure 3A).

In the middle stage of shoot emergence, the effects of light and nitrogen on Chls and Car contents remained consistent with the early stage. Nitrogen significantly influenced the Chl a/b ratio. Unlike in the early stage, the Chl a/b ratio was lowest under medium nitrogen treatment at 10% and 40% light intensity, but it increased as the nitrogen level rose under 100% light intensity (Figure 3B).

During the late shooting stage, the Chl a/b ratio was highest at 40% light intensity under low and medium nitrogen treatments. In high nitrogen treatment, the Chl a/b ratio increased as light intensity decreased (Figure 3C).

#### 3.2.2. Gas Exchange Parameters in Leaves During Shooting

**Figure 4:**
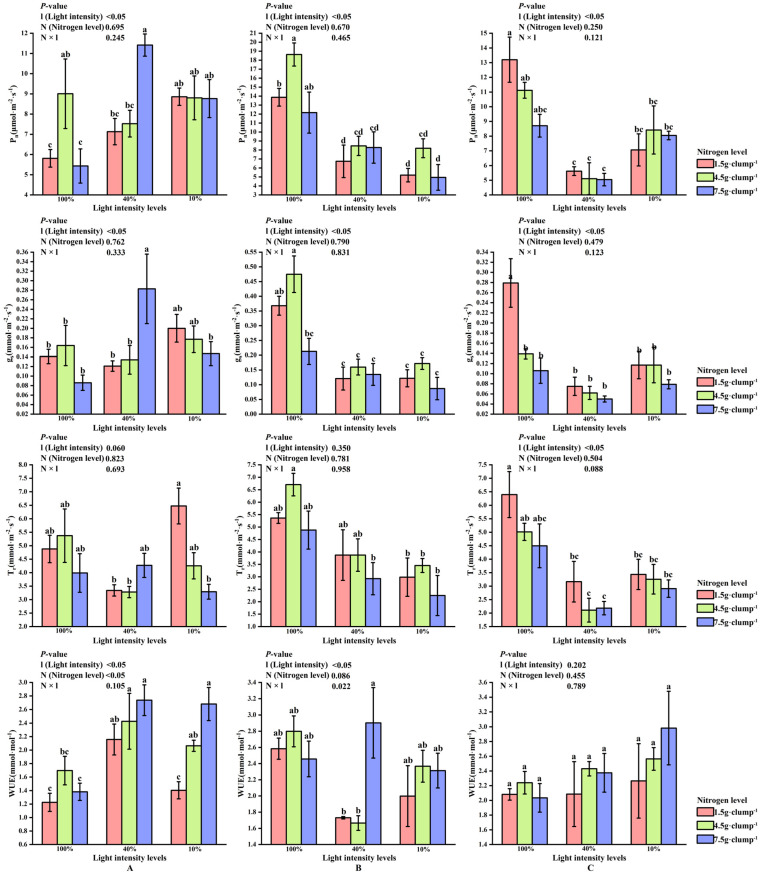
Interaction effects of light intensity and nitrogen concentration on gas exchange parameters in *Dendrocalamus latiflorus* seedlings. (Values are presented as means ± standard deviation (SD). Different letters indicate significant differences among nitrogen treatments within the same light intensity (one-way ANOVA, *p* < 0.05). (**A**), initial shooting phase; (**B**), intermediate shooting phase; (**C**), late shooting phase; 1.5 g·clump^−1^, nitrogen level was 1.5 g·clump^−1^; 4.5 g·clump^−1^, nitrogen level was 4.5 g·clump^−1^; 7.5 g·clump^−1^, nitrogen level was 7.5 g·clump^−1^. Abbreviations: P_n_, net photosynthetic rate; g_s_, stomatal conductance; T_r_, transpiration rate; WUE, water use efficiency).

At the early shooting stage, light intensity had significant effects on P_n_ and g_s_ (*p* < 0.05). Under the medium nitrogen treatment, both P_n_ and g_s_ were higher at 10% and 100% light intensities than at 40% light intensity (Figure 4A).

At the middle stage, neither light intensity nor nitrogen treatment significantly affected T_r_. In contrast, P_n_ and g_s_ were consistently higher under 100% light intensity than under 10% and 40% light intensities across nitrogen treatments (*p* < 0.05) (Figure 4B). Detailed results for Ci, which showed no significant responses to light or nitrogen treatments, are presented in Supplementary Figure S1.

At the late stage, WUE were not significantly influenced by light or nitrogen treatments. In contrast, across nitrogen levels, P_n_, g_s_, and T_r_ were highest under 100% light intensity and lowest under 40% light intensity. Under low nitrogen supply, P_n_, g_s_, and T_r_ at 100% light intensity were significantly higher than those at both 10% and 40% light intensities (*p* < 0.05) (Figure 4C).

#### 3.2.3. Chlorophyll Fluorescence Parameters in Leaves During Shooting

**Figure 5:**
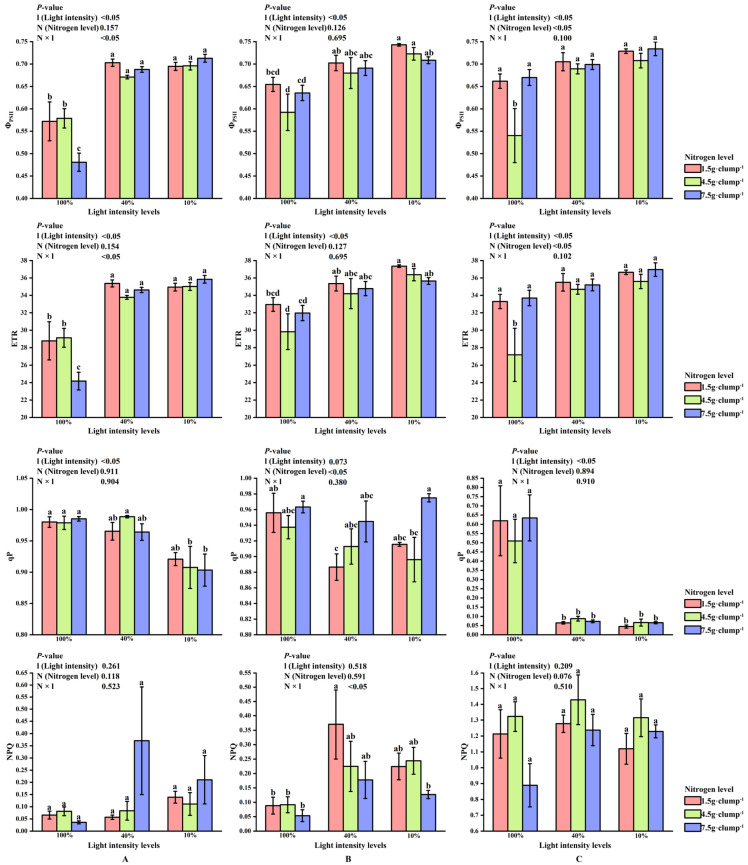
Interaction effects of light intensity and nitrogen concentration on chlorophyll fluorescence parameters in *Dendrocalamus latiflorus* seedlings. (Values are presented as means ± standard deviation (SD). Different letters indicate significant differences among nitrogen treatments within the same light intensity (one-way ANOVA, *p* < 0.05). (**A**), initial shooting phase; (**B**), intermediate shooting phase; (**C**), late shooting phase; 1.5 g·clump^−1^, nitrogen level was 1.5 g·clump^−1^; 4.5 g·clump^−1^, nitrogen level was 4.5 g·clump^−1^; 7.5 g·clump^−1^, nitrogen level was 7.5 g·clump^−1^. Abbreviations: Φ_PSII_, PSII actual photochemical efficiency; ETR, apparent photosynthetic electron transport rate; qP, photochemical quenching coefficient; NPQ, non-photochemical quenching coefficient).

At the early stage of shoot emergence, light intensity significantly affected Φ_PSII_, ETR, and qP (*p* < 0.05). Significant interaction effects between light and nitrogen treatments were detected for Φ_PSII_ and ETR (*p* < 0.05). Under the same nitrogen level, ΦPSII and ETR were significantly lower at 100% light intensity than at 10% and 40% light intensities (*p* < 0.05) (Figure 5A).

At the middle stage, photochemical performance showed sensitivity to high light intensity. Results for F_v_/F_m_, which exhibited relatively small variation across treatments, are provided in Supplementary Figure S2. Nitrogen treatment exerted a significant main effect on qP, while both light and nitrogen treatments, as well as their interaction, significantly affected NPQ (*p* < 0.05). Within the same light intensity, qP was highest under high nitrogen supply, and at 40% light intensity, qP increased with increasing nitrogen levels (Figure 5B).

At the late stage of shoot emergence, Φ_PSII_ and ETR were significantly lower under medium nitrogen treatment than under low and high nitrogen treatments at 100% light intensity (*p* < 0.05). Compared with the earlier stages, NPQ tended to increase while qP decreased across light and nitrogen treatments (Figure 5C).

#### 3.2.4. NSC Content in Leaves During Shooting

**Figure 6:**
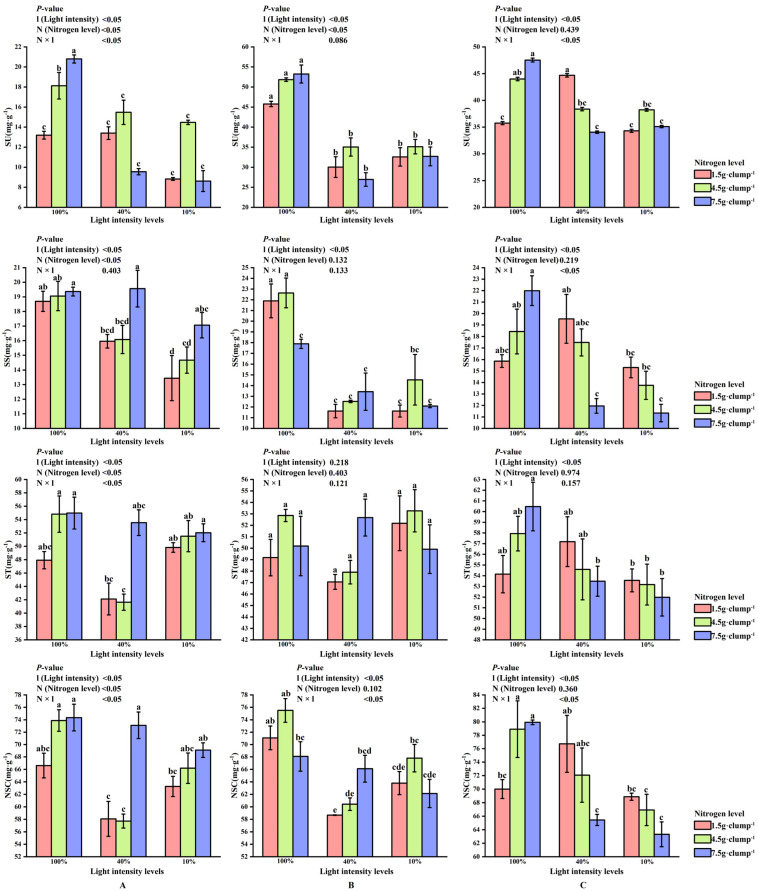
Interaction effects of light intensity and nitrogen concentration on NSC content of *Dendrocalamus latiflorus* seedlings. (Values are presented as means ± standard deviation (SD). Different letters indicate significant differences among nitrogen treatments within the same light intensity (one-way ANOVA, *p* < 0.05). (**A**), initial shooting phase; (**B**), intermediate shooting phase; (**C**), late shooting phase; 1.5 g·clump^1^, nitrogen level was 1.5 g·clump^−1^; 4.5 g·clump^−1^, nitrogen level was 4.5 g·clump^−1^; 7.5 g·clump^−1^, nitrogen level was 7.5 g·clump^−1^. Abbreviations: SU, sucrose; SS, soluble sugar; ST, starch; NSCs, nonstructural carbohydrates).

At the early shooting stage, both light and nitrogen treatments showed significant main effects, as well as a significant interaction effect, on leaf sucrose content (*p* < 0.05). Sucrose content was significantly higher under 100% light intensity than under 10% and 40% light intensities (*p* < 0.05) (Figure 6A). Under low and medium nitrogen treatments, starch and NSC contents at 40% light intensity were lower than those at 10% or 100% light intensity. In contrast, under high nitrogen treatment, starch and NSC contents decreased with decreasing light intensity.

At the middle stage, light intensity significantly affected soluble sugar and NSC contents (*p* < 0.05), and a significant interaction between light and nitrogen was detected for NSC content (*p* < 0.05), whereas starch content was not significantly affected by either factor (Figure 6B).

At the late shooting stage, light intensity significantly affected sucrose, starch, and NSC contents (*p* < 0.05). The SS/ST showed no consistent response pattern across treatments and is therefore presented in Supplementary Figure S3. Nitrogen treatment and the interaction between light and nitrogen also exerted significant effects on sucrose and NSC contents, whereas starch content was mainly influenced by light intensity, with no significant interaction effect detected (Figure 6C).

### 3.3. Carbon and Nitrogen Characteristics in Leaves During Shooting

**Figure 7:**
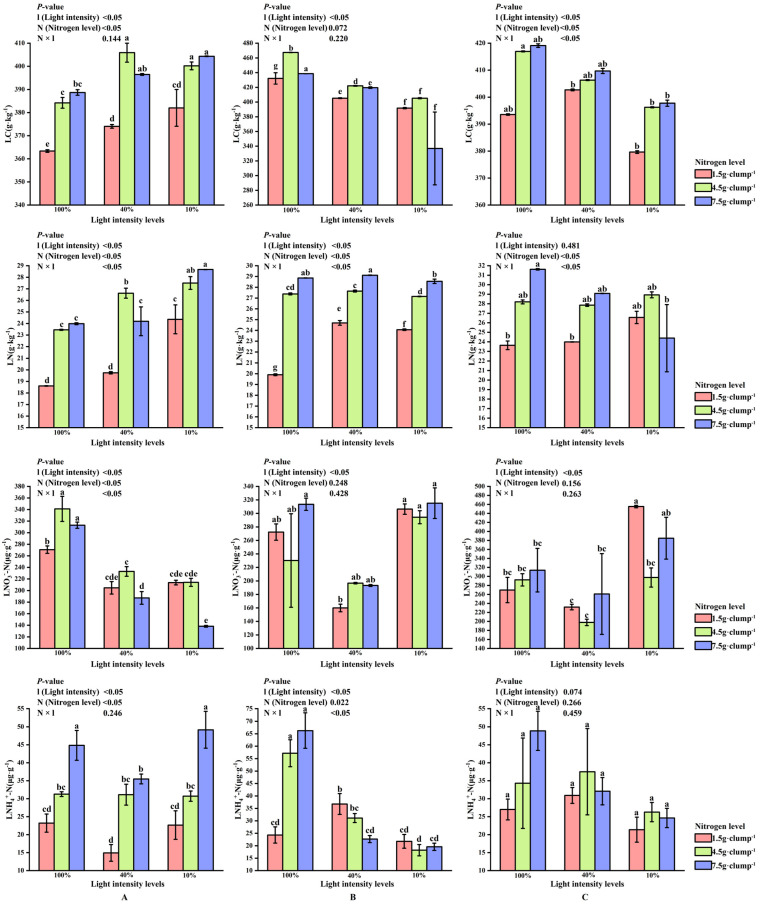
Interaction effects of light intensity and nitrogen concentration on leaf carbon and nitrogen characteristics of *Dendrocalamus latiflorus* seedlings. (Values are presented as means ± standard deviation (SD). Different letters indicate significant differences among nitrogen treatments within the same light intensity (one-way ANOVA, *p* < 0.05). (**A**), initial shooting phase; (**B**), intermediate shooting phase; (**C**), late shooting phase; 1.5 g·clump^−1^, nitrogen level was 1.5 g·clump^−1^; 4.5 g·clump^−1^, nitrogen level was 4.5 g·clump^−1^; 7.5 g·clump^−1^, nitrogen level was 7.5 g·clump^−1^. Abbreviations: LN, leaf total nitrogen; LC, leaf total carbon; LNO_3_^-^-N, leaf nitrate; LNH_4_^+^-N, leaf ammonium nitrogen).

At the early stage of shooting, both light intensity and nitrogen treatments had significant effects on LN and LC (*p* < 0.05). The LC/LN, which mainly reflected integrated carbon-nitrogen status and showed limited additional explanatory power, is presented in Supplementary Figure S4. A significant interaction effect between light and nitrogen was also observed for LC content (*p* < 0.05). Under 10% and 100% light intensities, LN and LC contents increased with increasing nitrogen levels. In contrast, under 40% light intensity, the highest LN and LC contents were recorded under medium nitrogen treatment (26.62 and 405.91 g·kg^−1^, respectively) (Figure 7A).

During the middle stage, the lowest LNO_3_^−^-N was consistently observed under 40% light intensity across all nitrogen treatments. Under low nitrogen supply, LNH_4_^+^-N was significantly higher at 40% light intensity than at 10% or 100% light intensity (*p* < 0.05). Under medium and high nitrogen treatments, LNH_4_^+^-N content was higher at 10% and 40% light intensities than at 100% light intensity (*p* < 0.05) (Figure 7B).

In addition, LN content increased with increasing nitrogen application across light treatments. Although light intensity significantly affected leaf LNO_3_^−^-N content *(p* < 0.05), no significant main effect of light intensity was detected for LNH_4_^+^-N content (Figure 7C).

### 3.4. Mechanism of Direct and Indirect Pathways Through Which Leaf Functional Traits on Bud Germination

**Figure 8:**
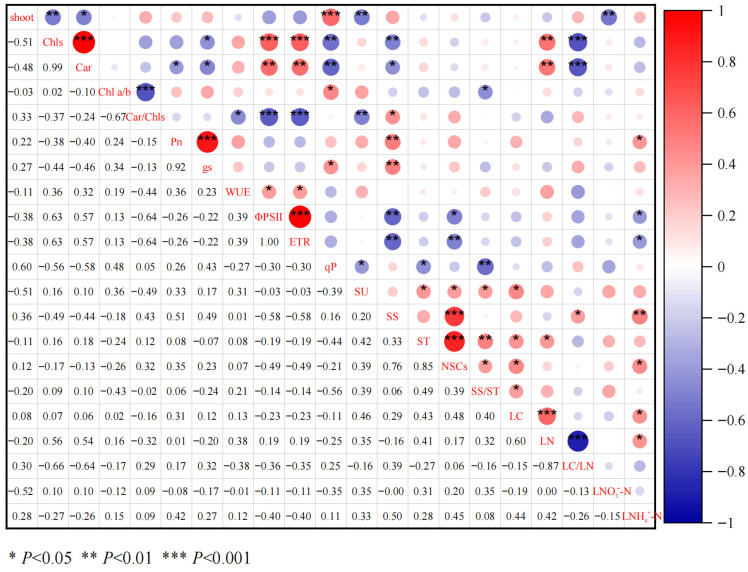
Pearson correlation among the influencing factors. (Abbreviations: shoot: number of emerging shoots; Chls, total chlorophylls; Car, carotenoids; Chl a/b, chlorophyll a/b; Car/Chls, carotenoids to total chlorophyll; P_n_, net photosynthetic rate; g_s_, stomatal conductance; WUE, water use efficiency; Φ_PSII_, PSII actual photochemical efficiency; ETR, apparent photosynthetic electron transport rate; qP, photochemical quenching coefficient; SU, sucrose; SS, soluble sugar; NSCs, nonstructural carbohydrates; SS/ST, soluble sugar /starch; LC, leaf total carbon; LN, leaf total nitrogen; LC/LN, leaf total carbon / total nitrogen; LNO_3_^−^-N, leaf nitrate; LNH_4_^+^-N, leaf ammonium nitrogen).

According to the correlation analysis (Figure 8), the number of germinated shoots was significantly negatively correlated with Chls, Car, SU, and LNO_3_^-^-N (*p* < 0.05), and significantly positively correlated with qP (*p* < 0.001). Chls was significantly positively correlated with Car, Φ_PSII_, ETR, and LN (*p* < 0.01), and Chls was significantly negatively correlated with g_s_, qP, SS, and LC/LN (*p*< 0.05). Car was significantly positively correlated with Φ_PSII_, ETR, and LN (*p* < 0.01), and Car was significantly negatively correlated with Pn, g_s_, qP, SS, and LC/LN (*p* < 0.05). qP was negatively correlated with SU, ST, and SS/ST (*p* < 0.05), and SU was positively correlated with ST, NSCs, SS/ST, and LC (*p* < 0.05).

**Figure 9:**
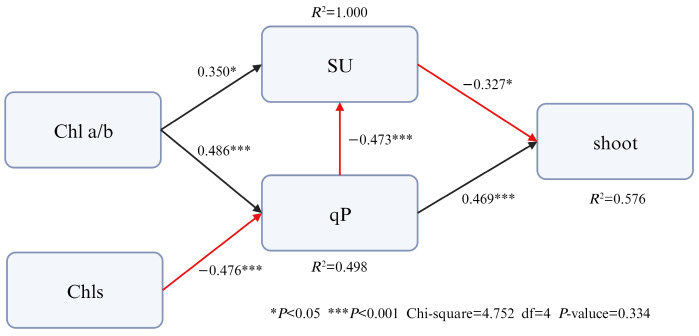
The direct and indirect pathways through which leaf functional traits of related factors on shoot germination of *Dendrocalamus latiflorus* seedlings. (Abbreviations: shoot: number of emerging shoots; SU, sucrose; qP, photochemical quenching coefficient; Chl a/b, chlorophyll a/b; Chls, total chlorophylls).

According to the results of Pearson correlation analysis, the functional trait indicators that have a significant impact on the number of shoot germinations were selected, and the structural equation model was constructed after eliminating the indicators with strong collinearity (Figure 9). The model fit the data well (1 < χ^2^/df = 1.188, *p* = 0.334). There was a significant positive correlation between qP and shoot germination (β = 0.469), and a significant negative correlation between SU and shoot germination (β = −0.327). qP also had an indirect effect on shoot germination by affecting SU (β = −0.473). Chl a/b indirectly affected the number of shoot bud germination by significantly affecting SU and qP; Chls indirectly affected the number of shoot germination by significantly affecting qP. These results indicate that qP is the most important functional trait affecting shoot bud number, working in concert with other indicators.

## 4. Discussion

### 4.1. Photosynthetic and Biochemical Characteristics

The interaction between nitrogen supply and light intensity has an important influence on the photosynthesis and physiological regulation of bamboo at the seedling stage. An appropriate amount of nitrogen fertilizer can promote chlorophyll synthesis, prolong leaf function period, and improve photosynthetic efficiency, thus providing continuous energy for shoot bud germination [22,23]. In the germination stage of *Dendrocalamus latiflorus* shoots, the addition of nitrogen fertilizer increased the contents of chlorophyll and carotenoid, with the highest values generally observed under moderate light conditions (40%), as indicated by the two-way ANOVA results. Plants can adapt to a low-light environment by enhancing light capture and energy conversion [24]. However, when the light intensity was too low (10%) and high nitrogen was applied, chlorophyll content tended to decrease, suggesting that the combination of low light and high nitrogen may limit the effective utilization of absorbed light energy and reduce photosynthetic efficiency.

Light intensity also plays an important role in regulating g_s_ and P_n_. Nitrogen plays a fundamental role in photosynthesis because a large proportion of leaf nitrogen is allocated to the photosynthetic apparatus, particularly to ribulose-1,5-bisphosphate carboxylase/oxygenase (Rubisco), which is the key enzyme responsible for CO_2_ fixation. Rubisco alone can account for 20–30% of total leaf nitrogen, making photosynthetic capacity highly sensitive to nitrogen availability [25]. Adequate nitrogen supply enhances Rubisco content and activity, thereby increasing carboxylation efficiency and net photosynthetic rate. In addition, nitrogen is a structural component of chlorophyll and proteins involved in the photosynthetic electron transport chain, further contributing to improved photochemical efficiency [26].

Based on the two-way ANOVA results, P_n_ and g_s_ reached their highest values under sufficient light (100%) with moderate nitrogen supply, suggesting maximized carbon fixation efficiency [27,28]. With decreasing light intensity, both gs and Pn declined synchronously, indicating reduced photosynthetic activity and assimilate production, which in turn constrained the energy supply required for shoot bud germination. Adequate nitrogen supply can partially alleviate the negative effects of low light by sustaining photochemical performance and carbon assimilation, thereby helping to maintain the growth potential of bamboo shoots [29,30].

In general, light and nitrogen supply jointly affected energy accumulation and physiological activity at the early stage of shoot germination by regulating photosynthetic efficiency and carbon assimilation. Strong light combined with appropriate nitrogen fertilizer can help to improve the supply of photosynthetic products and the growth rate of shoots, while low light or excessive nitrogen may lead to a decrease in photosynthetic efficiency, which is not conducive to the germination and early development of shoots.

### 4.2. The Regulatory Mechanism on Photosynthetic Products

Light intensity directly regulates carbon assimilation in leaves by modulating photosynthetic efficiency and electron transport, which in turn determines the amount and form of photosynthetic products accumulated in source organs. Under moderate light conditions, enhanced photochemical efficiency promotes sucrose synthesis and export, whereas low or excessive light may limit carbon assimilation or favor starch accumulation for photoprotection, thereby reducing carbon availability for shoot development [31,32].

Two-way ANOVA indicated that the interaction between light intensity and nitrogen supply (N × l) significantly affected the accumulation and distribution of bamboo photosynthetic products, thereby regulating the germination process of shoot buds. Carbohydrate produced by photosynthesis are the main energy source for shoot growth [33]. In the early stage of germination, moderate light (40%) and medium nitrogen levels can promote the coordinated accumulation of SS, SU, and ST, and provide stable energy for the development of new buds. However, under full light (100%) and high nitrogen treatments, excessive carbohydrate accumulation slightly inhibits P_n_ and g_s_, indicating that excessive light energy input may cause photoinhibition and limit carbon fixation [31].

As germination entered the middle stage, light intensity became the dominant factor affecting carbon metabolism. SS and NSCs decreased significantly under lower light (10–40%), as indicated by the two-way ANOVA results (*p* < 0.05), indicating that shading reduced photosynthetic yield and carbon skeleton supply, limiting new bud elongation and differentiation [34,35]. Nitrogen fertilizer application can partially alleviate this effect by increasing sucrose availability and promoting the effective utilization of non-structural carbohydrates, thereby providing a sufficient carbon source for bud development [36].

In the late stage of germination, high light combined with medium and high nitrogen treatment resulted in the highest accumulation of carbohydrates, which was significantly positively correlated with the number of new buds. This indicates that the coordinated supply of light and nitrogen promotes the energy supply and emergence rate of bamboo shoots by enhancing the carbon assimilation rate and photosynthetic product transport. In general, moderate light and nitrogen fertilizer levels can effectively balance the synthesis and utilization of photosynthetic products, optimize carbon allocation efficiency, and promote the germination and early growth of bamboo shoots.

### 4.3. Carbon and Nitrogen in Leaves During Shooting

The content of carbon and nitrogen in plant leaves is regulated by photosynthesis and nitrogen supply, which have an important influence on the germination of shoot buds [37]. In the early stage of germination, an appropriate amount of nitrogen can partially compensate for the carbon limitation caused by the decrease in photosynthetic efficiency under low light (10–40%) conditions, so that LC and LN can be maintained at a high level, thus providing the necessary energy and nutrients for the development of new buds [38]. In the middle stage, high nitrogen treatment significantly promoted nitrogen accumulation, which was positively correlated with the number of new buds, indicating that nitrogen supply was a key factor supporting bud growth [39].

In the late stage of germination, the synergistic effect of carbon and nitrogen further affected the germination performance. When the light was sufficient (100%) and the appropriate amount of nitrogen was applied, carbohydrate accumulation provided a substrate for nitrogen assimilation, improved the overall carbon metabolism efficiency, and thus supported the germination of shoot buds. In contrast, under low light or nitrogen deficiency conditions, carbon fixation was limited, and the number of germinated shoot buds decreased significantly [40,41]. In general, light and nitrogen affect the carbon source supply and nitrogen assimilation ability by regulating the carbon and nitrogen balance of leaves, thus directly regulating the germination rate and early growth potential of shoot buds.

### 4.4. Correlation Between Functional Traits of Leaves and Germination Number of Shoots

Pearson correlation analysis and path analysis were used to explore the statistical associations between leaf functional traits and the germination number of *D. latiflorus* shoots (Figure 8 and Figure 9). The results indicated that Chls, Chl a/b, qP, SU, and LNO_3_^-^-N exhibited relatively strong statistical associations with shoot germination under combined light and nitrogen treatments.

Chlorophyll is the fundamental photosynthetic pigment in green plants and reflects the photosynthetic potential of leaves [42]. Higher chlorophyll content is generally associated with greater photosynthetic capacity and assimilate production [43]. In the present analysis, both Chls and Chl a/b showed indirect statistical associations with shoot germination through their relationships with SU and qP (Figure 9). Variation in chlorophyll content and the Chl a/b ratio is commonly linked to adjustments in light utilization efficiency under different light environments [44,45].

The observed negative association between shoot number and pigment content likely reflects a trade-off in resource allocation between leaf-level light-harvesting investment and whole-plant growth processes. Under shaded or resource-limited conditions, increased pigment concentrations may represent a compensatory response to enhance light capture rather than increased carbon assimilation efficiency. In such cases, carbon gain and assimilate export remain constrained, limiting shoot initiation. Conversely, when light and nitrogen supply favor efficient carbon assimilation and assimilate transport, pigment concentrations may decrease due to dilution effects or reduced photoprotective demand, while more resources are allocated to shoot formation. Moreover, shoot number represents an integrative, whole-plant trait, whereas pigment content is a leaf-level trait, and this difference in organizational scale may further contribute to the observed negative relationship.

qP represents the proportion of absorbed light energy used for photochemical electron transport in PSII and is widely regarded as an indicator of photochemical efficiency [46,47]. Among the analyzed traits, qP exhibited the strongest statistical association with shoot germination. However, this association should be interpreted with caution, as correlation and path analyses identify potential relationships rather than causal or regulatory effects. The observed relationship between qP and shoot germination may reflect coordinated responses of photochemical performance and carbon assimilation under different light and nitrogen conditions. It should be noted that NPQ and related photochemical quenching parameters such as qP are highly plastic and rapidly regulated photoprotective traits, and relatively large variability among individuals represents a common physiological feature rather than experimental noise.

The Car/Chls ratio also showed a significant association with SU (Figure 8). Chlorophylls and carotenoids jointly participate in light harvesting and photoprotection processes [48]. Changes in the Car/Chls ratio are commonly associated with shifts in photoprotective investment and light energy utilization, which may influence the allocation of photosynthetic products between transportable carbohydrates and maintenance processes [32]. Leaf nitrate nitrogen (LNO3--N) was likewise associated with several photosynthetic traits and shoot germination, reflecting the close linkage between leaf nitrogen status, chloroplast development, photosynthetic enzyme abundance, and photochemical activity [49].

Overall, the results of correlation and path analyses suggest that multiple leaf functional traits are statistically associated with shoot germination under combined light and nitrogen treatments. These associations reflect coordinated physiological responses rather than definitive causal relationships, and further experimental or mechanistic evidence would be required to establish direct regulatory pathways.

## 5. Conclusions

This study demonstrates that the optimization of shoot emergence in *D. latiflorus* primarily depends on the balance between nitrogen supply and light intensity. Variations in light availability and nitrogen application jointly influenced shoot emergence, and imbalances between these two factors resulted in reduced shoot production. Photosynthetic traits, including chlorophyll characteristics, photochemical performance, and non-structural carbohydrate contents, exhibited close statistical associations with shoot emergence under different light and nitrogen conditions. These traits reflect the physiological responses of bamboo to environmental and nutritional conditions rather than acting as independent regulatory drivers of shoot emergence. Although qP showed the strongest association with shoot emergence in the present dataset, this result should be interpreted cautiously. The observed association does not imply a causal or dominant regulatory role of qP, but rather highlights its potential value as an indicator of photosynthetic performance under varying light–nitrogen regimes. Overall, the findings emphasize the importance of coordinated management of light conditions and nitrogen supply to enhance shoot emergence and early growth of *D. latiflorus*. It should be noted that the experiment was conducted under variable external environmental conditions, such as fluctuating temperature. Although these factors were not the focus of the present study, future research integrating physiological parameters with environmental variables may provide further insights into how environmental factors interact with physiological processes to regulate shoot germination.

## Figures and Tables

**Table 1:** The amount of nitrogen application per fertilization event.

Fertilization Times	N1 (1.5 g·clump^−1^)	N2 (4.5 g·clump^−1^)	N3 (7.5 g·clump^−1^)
I	0.45	1.35	2.25
II	0.60	1.80	3.00
III	0.45	1.35	2.25
Total amount	1.50	4.50	7.50

## Data Availability

The original contributions presented in this study are included in the article/Supplementary Materials. Further inquiries can be directed to the corresponding authors.

## Supplementary Materials

The following supporting information can be downloaded at: https://www.mdpi.com/article/10.3390/biology15010049/s1, Supplementary Figure S1. Interaction effects of light intensity and nitrogen concentration on C_i_; Supplementary Figure S2. Interaction effects of light intensity and nitrogen concentration on F_v_/F_m_; Supplementary Figure S3. Interaction effects of light intensity and nitrogen concentration on SS/ST; Supplementary Figure S4. Interaction effects of light intensity and nitrogen concentration on LC/LN.
