# Supplementary material for: Interactive Effects of Light Intensity and Nitrogen Supply on Shoot Emergence and Associated Photosynthetic Traits in Dendrocalamus latiflorus
Source: Biology (Basel). 2025 Dec 27;15(1):49. doi: 10.3390/biology15010049 (PMC12784682; doi:10.3390/biology15010049)
Supplement: Supplementary file 1 [file biology-15-00049-s001.zip › biology-4021721-supplementary.pdf]

Supplementary Materials

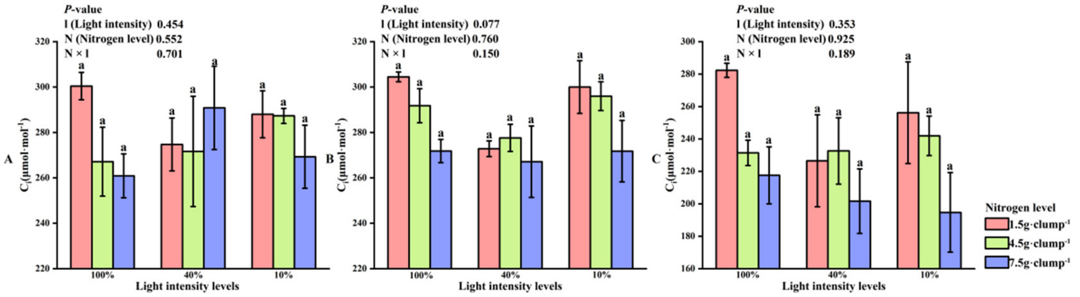

Supplementary Figure S1. Interaction effects of light intensity and nitrogen concentration on C_i

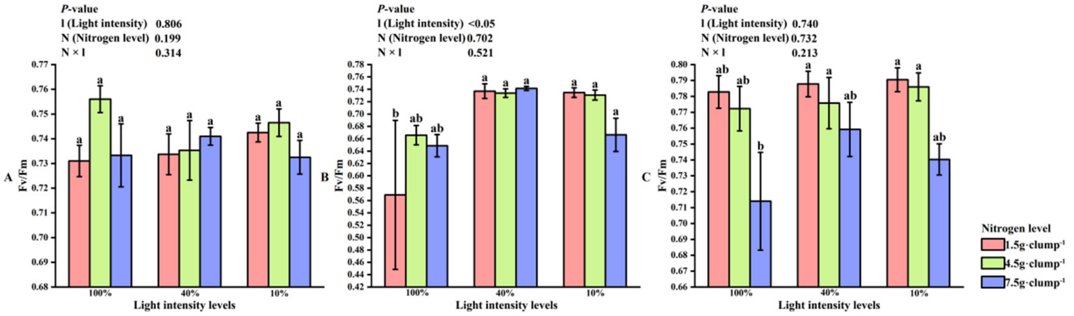

Supplementary Figure S2. Interaction effects of light intensity and nitrogen concentration on F_v/F_m

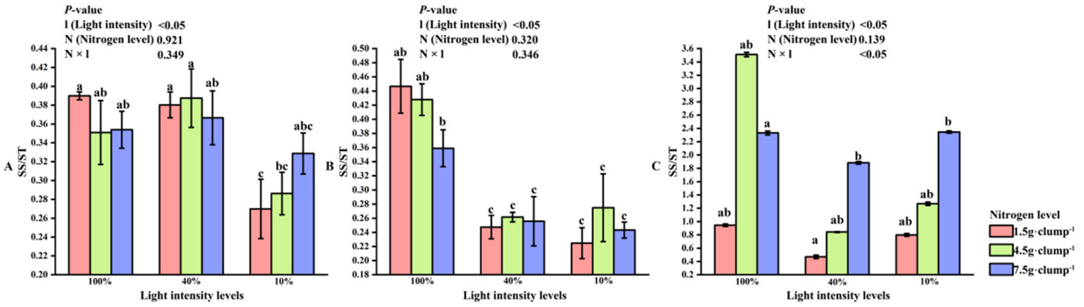

Supplementary Figure S3. Interaction effects of light intensity and nitrogen concentration on SS/ST

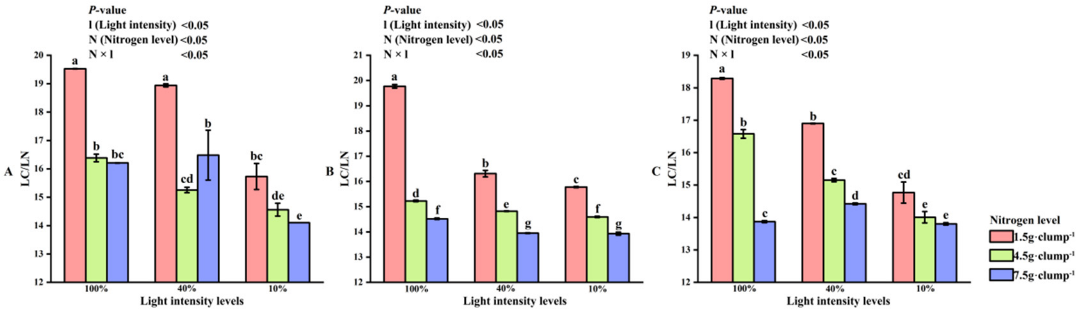

Supplementary Figure S4. Interaction effects of light intensity and nitrogen concentration on LC/LN
